# Reemergence of Oropouche Fever, Northern Brazil

**DOI:** 10.3201/eid1306.061114

**Published:** 2007-06

**Authors:** Raimunda do Socorro da Silva Azevedo, Márcio Roberto Teixeira Nunes, Jannifer Oliveira Chiang, Gilberta Bensabath, Helena Baldez Vasconcelos, Ana Yecê das Neves Pinto, Lívia Carício Martins, Hamilton Antônio de Oliveira Monteiro, Sueli Guerreiro Rodrigues, Pedro Fernando da Costa Vasconcelos

**Affiliations:** * Instituto Evandro Chagas, Belém, Pará, Brazil

**Keywords:** Oropouche fever, outbreaks, Brazilian Amazon, reemergence, dispatch

## Abstract

Oropouche fever has reemerged in Parauapebas and Porto de Moz municipalities, Pará State, Brazil. Serologic analysis (immunoglobulin M–ELISA) and virus isolation confirmed *Oropouche virus* (OROV) in both municipalities. Nucleotide sequencing of 2 OROV isolates from each location indicated genotypes I (Parauapebas) and II (Porto de Moz) in Brazil.

*Oropouche virus* (OROV), the cause of Oropouche fever, belongs to the family *Bunyaviridae*, genus *Orthobunyavirus,* Simbu serogroup (*1*), and is transmitted between humans in urban areas by the biting midge *Culicoides paraensis* (*2*,*3*). This virus was first isolated from febrile forest workers in Trinidad in 1955. The first isolation in Brazil was in 1960 from the blood of a sloth (*Bradypus tridactylus*) (*4*). The epidemic potential of OROV was recognized during an outbreak in Belém, Pará State, Brazil, in 1961, where ≈11,000 persons were infected (*4*). Over the past 45 years, many outbreaks of Oropouche fever, ≈500,000 cases, have been described in the Americas. OROV has been isolated in Trinidad, Panama, Peru, and Brazil, and in the past 40 years Oropouche fever has emerged as a public health problem in tropical areas of Central and South America (*3*).

Members of the genus *Orthobunyavirus* have a tripartite, single-stranded, negative-sense RNA genome of small (S), medium (M), and large (L) RNAs that encode nucleocapsid, glycoproteins, and RNA polymerase, respectively. Phylogenetic analysis of nucleocapsid genes of different OROV strains identified 3 distinct genotypes (I, II, and III) currently circulating in Central and South America; genotypes I and II have been detected in the Brazilian Amazon (*5*). Recently, an OROV isolate from a marmoset (*Callithrix* sp.) was characterized as a member of genotype III (*6*).

## The Study

**Figure 1:**
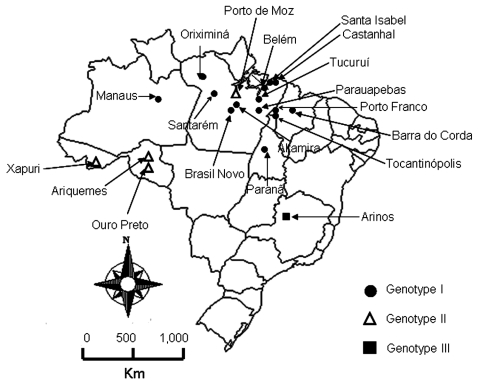
Map of Brazil showing locations where Oropouche fever outbreaks were identified during 2003–2004 and previous locations of this disease.

Two outbreaks of Oropuche fever occurred during 2003 and 2004. The first occurred in April–May 2003 in 2 communities (Vila Sansão, 140 inhabitants, and Vila Paulo Fontelles, 835 inhabitants).in the municipality of Parauapebas (6°4′S, 49°54′W). The second outbreak occurred in July–August 2004 in 1 community (Vila Tapara, 2,000 inhabitants) in the municipality of Porto de Moz (1°45′S, 52°14′W) (Figure 1).

A total of 125 and 109 serum samples were collected from residents of Parauapebas and Porto de Moz, which represented 12.8% and 5.45% of all inhabitants, respectively. Criteria for sampling were a history of acute fever several weeks before or during the survey or clinical symptoms similar to those of Oropouche fever. All serum samples were analyzed by hemagglutination inhibition (HI) test (*7*) and immunoglobulin M–ELISA (*8*) for specific HI and IgM antibodies to OROV. HI titers >20 and ELISA results greater than the cut-off value (optical density >0.200) were considered positive (*8*).Virus isolation was conducted by intracranial injection of newborn mice with a 1:10 (v/v) suspension of serum samples in phosphate-buffered saline, pH 7.4, as described elsewhere (*9*). Fifty-four and 11 serum samples from Parauapebas and Porto de Moz, respectively, were used for virus isolation. Identification of isolates was performed by complement fixation test as reported (*9*). Two OROV strains were isolated from patients in Parauapebas, and 2 strains were isolated from patients in Porto de Moz.

To genetically characterize the viruses, 2 isolates were selected from Parauapebas (Brazil 2003a and Brazil 2003b) and 2 from Porto de Moz (Brazil 2004a and Brazil 2004b). Viral RNA was extracted from Vero cells infected with human samples, and S RNA was amplified by using a 1-step reverse transcription–PCR assay as described (*5**,**6*). Phylogenetic trees were constructed for nucleocapsid gene nucleotide sequences by comparison with other OROV nucleocapsid gene sequences in GenBank (Table 1); neighbor-joining analysis (*10*) implemented in Mega version 2.1 (*11*) was used. Bootstrap analyses were performed on 1,000 replicates to generate confidence for groupings (*12*).

**Table 1:** Characteristics of *Oropouche virus* strains used for small RNA phylogenetic analyses

Strain	Source	Sample	Year	Location	GenBank strain identification	Accession no.
TRVL 9760	Human	Blood	1955	Trinidad	Trinidad 55	AF164531
BeAn 19991	*Bradypus trydactylus*	Blood	1960	São Miguel, Brazil	Brazil 60	AF164532
BeH 271815	Human	Blood	1975	Santarém, Brazil	Brazil 75	AF164533
BeAn 206119	*Bradypus trydactylus*	Blood	1971	Maracanã, Brazil	Brazil 71a	AY993909
BeAn 208402	*Bradypus trydactylus*	Blood	1971	Maracanã, Brazil	Brazil 71b	AY993910
BeAn 208819	*Bradypus trydactylus*	Blood	1971	Maracanã, Brazil	Brazil 71c	AY993911
BeAn 208823	*Bradypus trydactylus*	Blood	1971	Maracanã, Brazil	Brazil 71d	AY993912
BeH 390233	Human	Blood	1980	Manaus, Brazil	Brazil 80c	AF164536
BeH 381114	Human	Blood	1980	Belém, Brazil	Brazil 80b	AF164535
BeH 379693	Human	Blood	1980	Castanhal, Brazil	Brazil 80a	AF164534
BeH 472200	Human	Blood	1988	Porto Franco, Brazil	Brazil 88a	AF164537
BeH 472204	Human	Blood	1988	Tocantinópolis, Brazil	Brazil 88b	AF164538
BeAr 473358	*Culicoides paraensis*	Pool	1988	Porto Franco, Brazil	Brazil 88c	AF164539
BeH 475248	Human	Blood	1988	Tucuruí, Brazil	Brazil 88d	AF164540
GLM 444477	Human	Blood	1989	Panama	Panama 89a	AF164555
GLM 444911	Human	Blood	1989	Panama	Panama 89b	AF164556
GLM 445252	Human	Blood	1989	Panama	Panama 89c	AF164557
GLM 450093	Human	Blood	1989	Panama	Panama 89d	AF164558
BeH 505514	Human	Blood	1991	Santa Isabel, Brazil	Brazil 91a	AF164541
BeH 505442	Human	Blood	1991	Ouro Preto d’Oeste, Brazil	Brazil 91b	AF164542
BeH 505663	Human	Blood	1991	Ariquemes, Brazil	Brazil 91c	AF164543
IQT 1690	Human	Blood	1992	Peru	Peru 92	AF164549
MD 023	Human	Blood	1993	Peru	Peru 93a	AF164550
DEI 209	Human	Blood	1993	Peru	Peru 93b	AF164551
BeH 521086	Human	Serum	1993	Barra do Corda, Brazil	Brazil 93	AY704559
BeH 541863	Human	Blood	1996	Altamira, Brazil	Brazil 96a	AF164544
BeH 543033	Human	Blood	1996	Oriximiná, Brazil	Brazil 96b	AF164545
BeH 544552	Human	Blood	1996	Brasil Novo, Brazil	Brazil 96c	AF164546
BeH 543087	Human	Blood	1996	Xapuri, Brazil	Brazil 96d	AF164547
BeH 543618	Human	Blood	1996	Oriximiná, Brazil	Brazil 96e	AF164548
BeH 543733	Human	Serum	1996	Oriximiná, Brazil	Brazil 96f	AY704560
IQT 4083	Human	Blood	1997	Peru	Peru 97	AF164552
01–812–98	Human	Blood	1998	Peru	Peru 98a	AF164553
IQT 7085	Human	Blood	1998	Peru	Peru 98b	AF164554
BeAn 626990	*Callithrix sp*.	Viscera	2000	Arinos, Brazil	Brazil 00	AY117135
BeH 622544	Human	Blood	2002	Paranã, Brazil	Brazil 02	EF467368
BeH 669314	Human	Blood	2003	Parauapebas, Brazil	Brazil 03a	EF467370
Be H 669315	Human	Blood	2003	Parauapebas, Brazil	Brazil 03b	EF467369
BeH 682426	Human	Blood	2004	Porto de Moz, Brazil	Brazil 04a	EF467377
BeH 682431	Human	Blood	2004	Porto de Moz, Brazil	Brazil 04b	EF467372

Of 125 serum samples from patients in Parauapebas, HI results were positive for 16 (12.7%) from Vila Sansão, 6 (4.8%) from Paulo Fontelles, and 4 (3.2%) from other localities. IgM was detected in 16 (12.7%), 8 (4.8%), and 6 (4.8%) serum samples from these 3 areas, respectively. Of 117 serum samples from patients in Porto de Moz, 56 (46.7%) had HI antibodies and 61 (52.1%) had IgM to OROV.

A total of 71.9% of female patients in Parauapebas and 59% in Porto de Moz had symptoms suggestive of Oropouche fever. Although all age groups were affected, persons 5–14 years of age had the highest frequency of symptoms (30.4%) and those <1–4 years of age had the lowest frequency (4.8%) (Table 2). Symptoms most frequently reported were fever (100%), headache (79.3%), joint pain (68.7%), and muscle pain (30%). Seventy percent of patients reported ≥1 episode of recurrence of fever, characterized by fever, headache, and other symptoms ≈2–3 weeks after onset of initial symptoms (*2*,*3*).

**Table 2:** Distribution of serum samples positive for immunoglobulin M to *Oropouche virus* in 2 municipalities, Pará State, Brazil, 2003–2004

Patient age, y	Porto de Moz, no. positive/no. tested		Parauapebas, no. positive/no. tested	
	Male	Female	Male	Female
<1–4	1/6	3/4	0/2	1/9
5–14	11/21	7/19	3/21	7/24
15–24	2/7	7/14	0/4	4/13
25–34	4/7	6/10	0/3	5/11
35–44	4/5	3/5	4/4	1/7
45–54	2/3	4/8	1/7	3/8
≥55	2/3	5/5	1/6	2/5
Total	26/52	35/65	9/47	23/77

**Figure 2:**
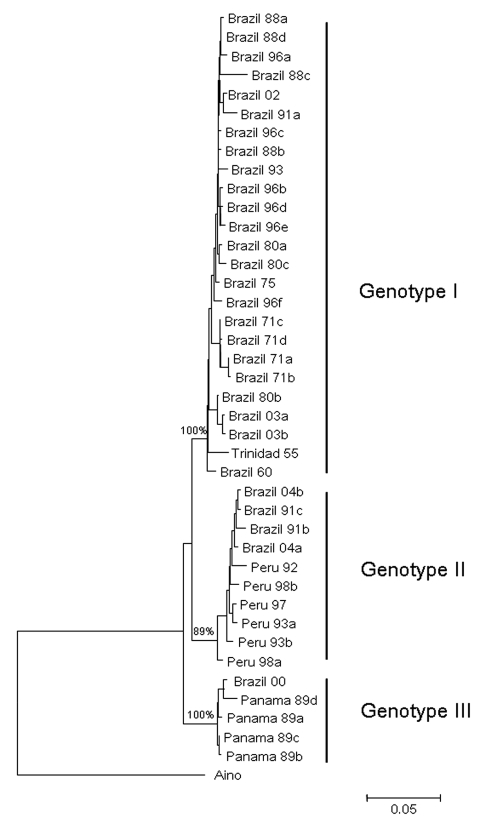
Comparative small (S) RNA phylogenetic tree constructed by using the neighbor-joining method for *Oropouche virus* strains isolated in Parauapebas and Porto de Moz, Pará State, Brazil. Bootstrap values were placed over the 3 nodes for each main group (I, II, and III). Aino virus S RNA sequence was used as an outgroup. Scale bar indicates a divergence of 5% in the nucleotide sequence.

Full-length S RNA of the 4 OROV strains contained 754 nt and encoded 2 overlapping open reading frames, the nucleocapsid (693 nt and 231 aa) and nonstructural protein (273 nt and 91 aa). Two small noncoding regions were also found at the 3′ and 5′ ends of these reading frames, spanning nt positions 1–44 and 741–754, respectively. Phylogenetic analysis of Brazil 2003 and 2004 isolates grouped strains from Parauapebas (Brazil 2003a and Brazil 2003b) into OROV genotype I and strains from Porto de Moz (Brazil 2004a and Brazil 2004b) into OROV genotype II (Figure 2).

## Conclusions

Oropouche fever is the second most common arboviral disease (after dengue fever) in the Brazilian Amazon region. From 1960 to 1980, Oropouche fever outbreaks were detected only in Pará State, mainly in Belém and neighboring areas, where thousands of people were infected (*2*,*3*). OROV was then detected in other Amazonian states including Amazonas, Amapá, Acre, Rondônia, and Tocantins; and non-Amazonian states, including Maranhão in northeastern Brazil and Tocantins in central Brazil (*3*,*8*). Recently, OROV isolated from *Callithrix* sp.in Arinos, Minas Gerais State, southeastern Brazil was characterized as genotype III, which indicated the presence of this genotype in Brazil (*6*). OROV from this species has been identified only in Panama (*5*). From 1980 to 2005, sporadic cases or self-limited outbreaks of Oropouche fever were reported in areas of the Brazilian Amazon, which suggested silent endemic circulation of the virus (*13*). In 2003 and 2004, several cases of Oropouche fever were detected in Parauaebas and Porto de Moz in Pará State. Parauaebas is located in the Carajás mineral province and Porto de Moz is located in the Altamira region.

Genetic characterization of strains indicated the presence of genotype II in the eastern Amazon region. This genotype had been associated with cases of Oropouche fever in restricted western Amazonian areas (Rondônia State), as well as in Peru (*5*). This finding suggests movement of OROV genotype II across the Amazon region from western to eastern areas or emergence of this genotype after silent circulation for several years. Genotype I (Brazil 2003a and Brazil 2003b) found in Parauapebas was closely related to Trinidadian and Brazilian isolates obtained from 1955 through 1960 (Trinidad 55 and Brazil 60) (*5*). Genotype II strains isolated in Porto de Moz were genetically related to strains isolated in Peru during the 1990s (Peru 92, 93, 97, 98a, 98b) and Rondônia State in 1991 (Brazil 91a, 91b), as reported by Saeed et al. (*5*). These data indicate that Parauapebas and Porto de Moz OROV isolates are genetically distinct and have different ancestor viruses (Figure 2). Recognition of different OROV genotypes in the Brazilian Amazon, as well as new genetic information, is useful for understanding the epidemiology and genetic diversity of this emergent human pathogen.
